# Effects of acute ebselen add-on treatment on resting state function connectivity in depressed patients with inadequate response to antidepressants

**DOI:** 10.1038/s41598-026-58393-2

**Published:** 2026-06-20

**Authors:** Fitri Fareez Ramli, Luca M. Villa, Marieke A. G. Martens, Nisha Singh, Catherine J. Harmer, Philip J. Cowen, Beata R. Godlewska

**Affiliations:** 1 Psychopharmacology Research Group, Department of Psychiatry, University of Oxford, Warneford Hospital, Oxford, OX3 7JX UK; 2 Oxford Health NHS Foundation Trust, Oxford, UK; 3 Department of Pharmacology, Faculty of Medicine, Universiti Kebangsaan Malaysia, Kuala Lumpur, 56000 Malaysia; 4 QYNAPSE SAS, 2-10 Rue d’Oradour-sur-Glane, Paris, 75015 France; 5 Department of Paediatrics, University of Oxford, Oxford, OX3 9DU UK

**Keywords:** Antidepressant, Depression, Ebselen, Lithium-mimetic, Diseases, Neurology, Neuroscience

## Abstract

Ebselen has been shown to induce a positive emotional processing bias and brain neurochemistry in healthy volunteers. However, little is known about its effects on resting-state functional connectivity (rs-FC) in depressed patients who show inadequate responses to standard antidepressants. The study aimed to investigate the effect of ebselen as an add-on treatment on rs-FC in this population. We conducted a randomised, double-blind, placebo-controlled trial involving depressed patients who were not responsive to their current treatment. Participants received either ebselen 600 mg twice daily or placebo for seven days. Resting-state functional magnetic resonance imaging was conducted at baseline and on day seven. Resting-state data were analysed using seed-based analysis and independent component analysis. Greater changes in rs-FC between the pregenual anterior cingulate cortex and the cerebellum were observed in ebselen than in placebo, but became negligible after corrections for multiple comparisons. We identified treatment effects within visual networks and between visual and sensorimotor networks, however, these did not survive correction for multiple comparisons. Short-term ebselen treatment in depressed patients with inadequate antidepressant responses did not produce significant changes in rs-FC. These findings may reflect limitations related to the short treatment duration and small and heterogeneous sample.

## Introduction

Lithium is an effective treatment for bipolar disorder and treatment-resistant depression. However, its use has declined in recent years, partly due to associated adverse effects, including thyroid dysfunction and renal impairment. In addition, its narrow therapeutic index necessitates regular monitoring, which may further reduce its preference among patients and prescribers. Therefore, there is a need to identify lithium-mimetic agents that can retain its therapeutic benefits while minimising the risk of adverse effects.

In the search for a lithium-mimetic agent that possesses the therapeutic benefits of lithium while minimising its adverse effects, ebselen has emerged as a promising candidate due to its similar mechanisms of action to lithium. A growing body of preclinical and clinical research in mood disorders has established a strong mechanistic foundation supporting the potential use of ebselen in bipolar disorder and treatment-resistant depression^1–7^.

Ebselen has been shown to reduce myo-inositol levels potentially through inhibition of inositol monophosphatase (IMPase) in both preclinical models and human experimental studies^1,2,4–7^. IMPase is a key enzyme within the phosphoinositide signalling pathway, a second messenger system utilised by several serotonergic receptors (5-HT), including 5-HT_2A_ and 5-HT_2C_^4^. Both ebselen and lithium have shown to induce behavioural and molecular changes associated with 5HT_2A_ agonists^4,7^. Interestingly, ebselen also has been shown to attenuate molecular changes induced by the 5HT_2C_ agonist^8^, suggesting complex modulatory roles within serotonergic signalling and requirefurther investigation.

In human studies involving healthy volunteers, ebselen administration reduced myo-inositol in the anterior cingulate cortex (ACC), a key brain region involved in emotion processing. These neurochemical changes coincided with a positive shift in emotion processing, suggesting a potential use of ebselen as a mood-regulating agent in mood disorders^1,5,6^.

Functional magnetic resonance (fMRI) imaging provides valuable mechanistic insights into the neural underpinnings of psychiatric disorders and their treatment. One variation of fMRI is resting-state fMRI, which enables an assessment of functional connectivity, a temporal correlation of the activity of brain regions, when an individual is awake yet not engaged in any external tasks. Patients with depression who are resistant to multiple treatments have been shown to exhibit altered resting-state functional connectivity within the affective and cognitive control networks compared with treatment-responsive counterparts^9^. Notably, ketamine treatment in patients with treatment-resistant depression has been associated with normalisation of hyperconnectivity within the default mode network, a core network implicated in maladaptive self-referential processing and rumination. Collectively, these findings suggest that alterations or normalisation of resting-state functional connectivity may provide mechanistic insight into how pharmacological interventions exert therapeutic effects in depression^10–12^.

To our knowledge, no previous studies have examined the effects of ebselen on resting-state functional connectivity in a clinical population. Therefore, the present study aimed to investigate the effects of acute ebselen administration as an add-on treatment on resting-state functional connectivity in depressed patients with inadequate responses towards standard antidepressants. A secondary objective was to explore the association between changes in neurometabolite concentrations of myo-inositol, glx, and glutamate in the ACC and alterations in resting-state functional connectivity between the ebselen and placebo groups.

## Methods

We conducted a double-blind, randomised, placebo-controlled, experimental trial involving patients with major depressive episodes who were still depressed despite adequate duration and dose of antidepressants. This study was a part of the larger study reported before (for more details, refer to^13^. The study was approved by the National Research Ethics Service Committee [NRES], South-Central Oxford (D:20/SC/0151). All methods were performed in accordance with the relevant guidelines and regulations. All participants gave their written informed consent before the study.

The present fMRI analysis was conducted as part of a larger study in which the primary outcome was emotional processing. The a priori sample size calculation was therefore based on the expected effect of short-term antidepressant treatment on the emotional face recognition task. Based on the effect size reported by Harmer and colleagues^14^ following 7 days’ antidepressant treatment on facial expression recognition, a sample size of 25 participants receiving active treatment was estimated to provide 90% power to detect a similar effect size of approximately 0.4, using an alpha level of 0.05. The original target sample size was therefore approximately 25 participants per treatment group.

We screened participants using the Structured Clinical Interview for Diagnostic and Statistical Manual of Mental Disorders (DSM-5) (SCID-5). We included patients with major depressive disorder and a Hamilton Depression Rating Scale score of *≥* 14 who had inadequate responses to their current antidepressant treatment. The participants were required to have been on their current antidepressant at a therapeutic dose for at least four weeks. The participants were excluded if they had: history of or current psychosis, bipolar disorder and emotionally unstable personality disorder; history of significant substance dependence (including alcohol) over the past six months; clinically significant risk of suicide; current or history of electroconvulsive therapy for the current depressive episode; failure to respond to standard pharmacological augmentation treatments (lithium and atypical antipsychotics); severe claustrophobia; body mass index outside the range of 18–36 kg/m^2^; contraindications to magnetic resonance imaging scanning; current pregnancy and lactation; participation in a study involving emotional processing tasks or interventional medication within the last three months. We did not exclude the participants with medical conditions on prescribed medications if these did not compromise the safety or affect data quality. Premenopausal women and male participants engaging in sexual activity with a risk of pregnancy were required to use highly effective contraception during the study and for 30 days after receiving the study medication. Male participants were required not to donate sperm during this period.

Participants were randomised to receive ebselen 600 mg or placebo twice daily for seven days in a 1:1 allocation. Randomisation was stratified by sex and number of prior antidepressant treatments, generating four strata: male with 1–2 prior antidepressant treatments, male with > 2 prior antidepressant treatments, female with 1–2 prior antidepressant treatments, and female with > 2 prior antidepressant treatments. Within each stratum, participants were allocated sequential subject numbers as they entered the study, according to the pre-specified randomisation schedule. The MRI scans (structural, proton magnetic resonance spectroscopy (MRS) and functional MRI) were conducted at baseline and on day seven of treatment.

### Data acquisition

The structural, MRS and resting-state functional MRI (fMRI) scans in that order were conducted at the Oxford Centre for Human Brain Activity, University of Oxford, using 3 T SIEMENS MAGNETOM with a 32-channel receive array head coil (Siemens, Erlangen, Germany). Structural images were acquired using a T1- Magnetization Prepared - RApid Gradient Echo (MPRAGE) image with a voxel resolution of 1.0 × 1.0 × 1.0 mm with a repetition time (T_R_) = 1900 ms, echo time (T_E_) = 3.96 ms, flip angle = 8^o^, slice thickness = 1 mm, 192 slices, field-of-view (FoV) read = 256 mm). A detailed description of proton MRS pre-processing can be found in another publication (refer to^13^. Briefly, an 8-ml voxel was manually placed in the ACC of the MPRAGE image acquired^15^. The adjustments of first- and second-order shims were conducted using gradient echo shimming^16^. We acquired the spectra using the semi-Localization by Adiabatic Selective Refocussing sequence (T_E_ = 28 ms, T_R_ = 3 s, 128 averages) with variable power radiofrequency pulses with optimized relaxation delay (VAPOR)^17^.

Before the resting-state functional MRI scan, participants were instructed to look at the fixation mark in the centre of the white background and reminded to stay awake during the fMRI scan (voxel size of 2.4 × 2.4 × 2.4 mm, T_R_ = 735 ms, T_E_ = 39 ms, flip angle = 52^o^, slice thickness = 2.4 mm, 192 slices, FoV read = 210 mm, 800 volumes, and 64 slices). Gradient echo phase and magnitude field maps were also acquired for correcting any distortions (2.4 × 2.4 × 2.4 mm, T_R_ = 600 ms, T_E1_/T_E2_: 4.50/6.96 ms, flip angle = 46^o^).

### Resting-state fMRI pre-processing and statistical analysis

The data were pre-processed and analysed using FSL (version 6.6.1, FMRIB Analysis Group, University of Oxford, UK)^18^. Individual subject data were pre-processed (brain extractions of T1-MPRAGE and fieldmap magnitude images, creations of fieldmap images). Further, the Multivariate Exploratory Linear Optimized Decomposition into Independent Components (MELODIC, version 3.15) function in FSL was used to perform the individual subject independent component analysis (ICA), including motion correction, unwarping of effective echo-planar imaging data (EPI echo spacing = 0.64 ms, EPI T_E_ = 39, unwarp direction = -y, percentage signal loss threshold = 10), co-registration of EPI data to the structural scan using boundary-based registration, and normalization into Montreal Neurological Institute (MNI) standard space using both linear and non-linear registrations (warp resolution = 10 mm, resampling resolution = 2 mm)^19^. We also applied temporal filtering with a high-pass filter cut-off of 100 s (0.005 Hz), spatial smoothing with a Gaussian kernel of full-width at half-height (FWHM) of 5 mm, variance time course, automatic dimensionality estimation and IC map threshold of 0.5. We performed a data quality check on the first-level analysis. The following criteria were used to indicate acceptable motion: movement *≤* 2.4 mm in translation along x, y, or z planes and *≤* 3^o^ (equivalent to 0.052 radians] rotation in any direction [pitch, roll or yaw)^20^.

The study-specific training dataset was created by manually labelling ten randomly selected individual datasets into signal and noise^21^. The Functional MRI of the Brain (FMRIB)’s ICA-based Xnoiseifier (FIX) was used to train the study-specific dataset. We selected the best threshold of 20, with a true positive ratio of 97.3 and a true negative ratio of 89.9, to denoise all data. Subsequently, seed-based functional connectivity analysis and independent component analysis were conducted separately. For seed-based analyses, the pregenual ACC was chosen as a seed due to the significant effects of ebselen in reducing myo-inositol and glx (a composite measure of glutamate+glutamine) levels in the ACC which coincided with a positive shift in emotional processing^22,23^. All seed-based analyses investigated the functional connectivity between the seed region and the whole brain. In line with these previous studies, our MRS data, used for association testing in this study, were acquired from a voxel located in the ACC^13^. Also, increased connectivity between the ventromedial prefrontal cortex (of which ACC is a part) and the amygdala was associated with clinical improvement in bipolar mood disorder patients receiving lithium monotherapy, indicating that ACC might be sensitive to the effect of a lithium-mimetic agent ebselen^24^. The pregenual ACC mask was created from Automated Anatomical Labelling atlas 3^25^. Dual regression was conducted to produce subject-specific timeseries and spatial maps^26–28^.

FSL voxel-based morphometry (FSLVBM) was used for quantifying grey matter volumes, which were included as a covariate for resting-state functional connectivity analyses^29–31^.

The general linear model was used to compare the changes (post-treatment minus baseline parameters) in functional connectivity between ebselen and placebo with grey matter volumes included as a covariate. All analyses of resting-state functional connectivity utilised FSL randomise to analyse the data using permutation-based analysis (number of permutations = 5000) using threshold-free-cluster-enhancement (TFCE) and a family-wise error (FEW) corrected p-value threshold of < 0.05^31–33^.

The whole brain resting-state fMRI analysis (group ICA) was also conducted to determine changes in resting-state functional connectivity across the whole brain. We utilised the MELODIC to temporally concatenate the de-noised functional data and decompose them into independent components of 25. Spatial mixture modelling estimates that the average number of individual IC maps is typically about 25^27^. The potential networks were identified by visual inspections. The correlation between the identified network and published resting state networks^27,34^ was analysed using the FSL cross-correlation tool (threshold of 0.3). Dual regression was conducted to produce subject-specific timeseries and spatial maps^26–28^. Similarly, the permutation-based analysis (number of permutations = 5000) as described above was conducted with grey matter volumes as a covariate^31–33^. Parameter estimates of changes in resting-state functional connectivity measures were extracted for all significant clusters.

### Proton magnetic resonance spectroscopy pre-processing and analysis

The LCModel in MATLAB 2016 was used to measure concentrations of myo-inositol, glx, and glutamate, with total creatine (creatine and phosphocreatine) as reference^35^. The cerebrospinal fluid, white matter, and grey matter percentages were measured to correct metabolite concentrations^36^.

The correlation between changes in neurometabolite concentrations (myo-inositol, glx and glutamate) in the ACC and changes in resting-state functional connectivity between the pregenual ACC and other brain regions were conducted using permutation-based analysis, with metabolite levels as a covariate.

### Statistical analysis for patient baseline characteristics

Descriptive data were presented as means ± standard error of means (SEMs) for continuous data and count and percentages for categorical data. Independent t-tests and chi-square tests were used to analyse continuous and categorical data, respectively.

## Results

Following randomisation, 55 participants were allocated to treatment, with 28 assigned to the ebselen group and 27 to the placebo group. rs-fMRS data were unavailable for 10 participants for the following reasons: no fMRI scan was acquired due to safety concerns (*n* = 3) or lack of scanner availability within the treatment window (*n* = 1); an adverse reaction during scanning (*n* = 1); COVID-19 infection (*n* = 1); non-compliance (*n* = 1); and exclusion of data due to excessive motion artefacts (*n* = 3).

Forty-five participants aged 18–66 (37.1 *±* 2.0 years) were included in the final analysis, of whom 25 were allocated to the ebselen group and 20 to the placebo group (Table 1). There were no significant differences between groups in age (*t*(43) = 0.007, *p* = 0.994), sex (*p* = 0.371 (*χ* ^2^)), number of antidepressant treatments (*p* = 0.944 (*χ* ^2^)) and baseline HAM-D (*t*(43) = 0.213, *p* = 0.833). Please see Table 1 for details.

**Table 1:** Sociodemographic and clinical data.

Characteristic	Ebselen group, *n* = 25	Placebo group, *n* = 20	Total, *n* = 45	Statistical tests (ebselen vs. placebo groups)
Sex, n				*p* = 0.371 (χ 2)
Male	8	9	17	
Female	17	11	28	
Age, mean *±* SEM (years)	37.1 *±* 2.4	37.1 *±* 3.4	37.1 *±* 2.0	t(43) = 0.007, *p* = 0.994
Number of prior antidepressant treatments, n				*p* = 0.944 (χ 2)
1–2 prior treatments	16	13	29	
>2 prior treatments	9	7	16	
Baseline depression severity, mean *±* SEM	19.44 *±* 0.99	19.15 *±* 0.89	19.31 *±* 4.50	t(43) = 0.213, *p* = 0.833

Three participants were excluded due to significant movements of > 2.4 mm in translation along x, y, or z planes or 3^o^ (equivalent to 0.052 radians) rotation in any direction (pitch, roll, or yaw)^19^. The mean framewise displacement was 0.14 mm, SD = 0.08, in the ebselen group and 0.14 mm, SD = 0.07, in the placebo group (t = 0.161, *p* = 0.872).

### Seed-based analysis of changes in resting-state functional connectivity between pregenual ACC and other brain regions and its correlations with neurometabolite changes in ACC

**Fig. 1:**
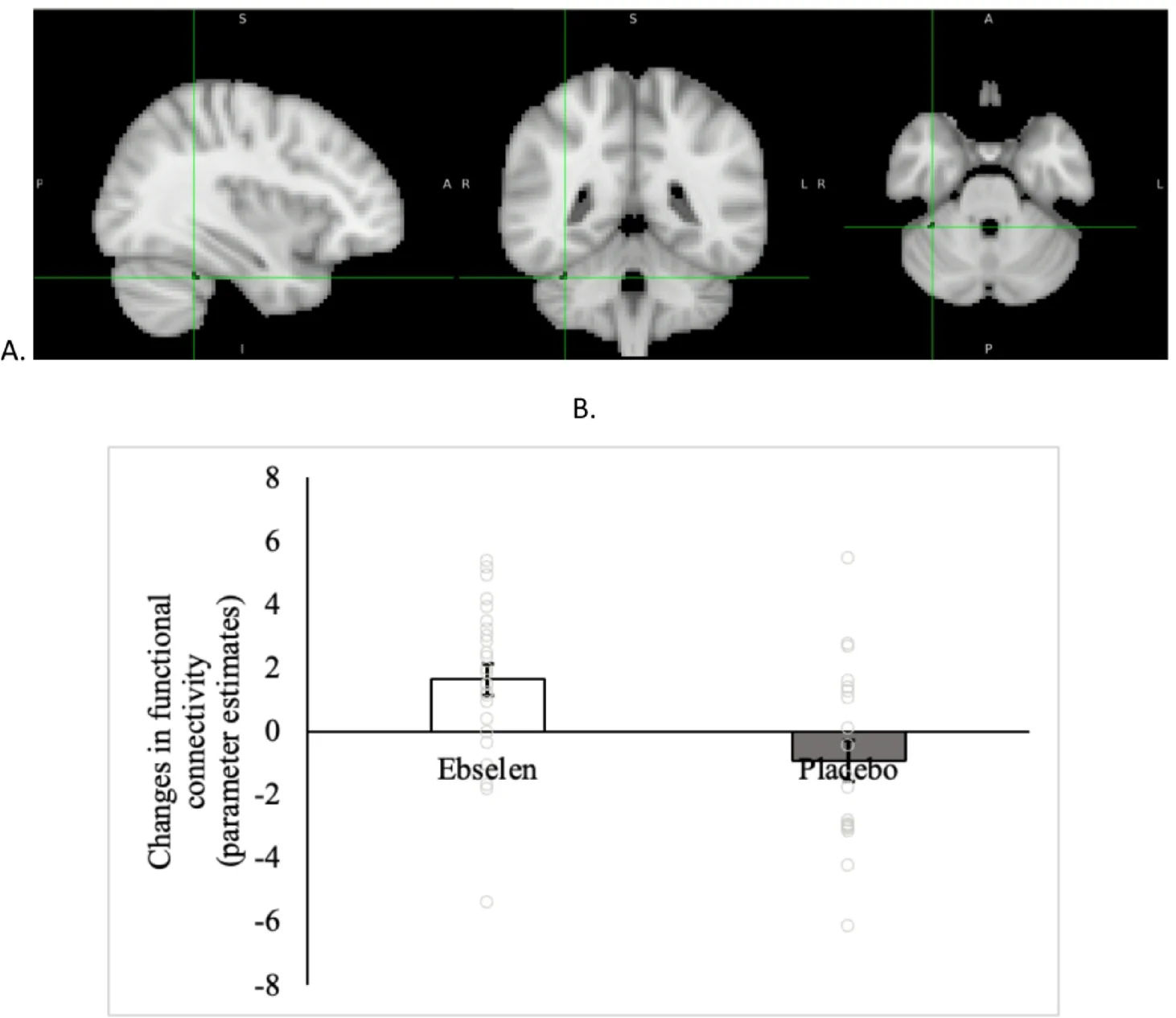
Changes in resting-state functional connectivity between the pregenual ACC and the cerebellum over treatment period. **(A)** Darker voxels in the cerebellum (MNI 27, 41, 21) indicate greater increase in resting-state functional connectivity with pregenual ACC in the ebselen group than in the placebo group. Results are reported using TFCE method, with clusters considered significant at a threshold of *p* < 0.05. Cluster size = 4 significant voxels, uncorrected peak p-value − 0.041, t = 6.56. The result was not significant after correction for multiple comparisons (2 x *t-test*, corrected significant *p* < 0.025). **(B)** Parameter estimates of the changes in resting-state functional connectivity between pregenual ACC and cerebellum in both groups.

Group comparisons, using TFCE method, with clusters considered significant at a threshold of *p* < 0.05, showed a greater increase in resting-state functional connectivity between the pregenual ACC and the right cerebellum (number of significant voxels = 4, MNI 27, 41, 21, uncorrected peak p-value 0.041, Hedge’s g = 0.943 (large effect size) in the ebselen group but became non-significant after correction for multiple comparisons (2 t-tests comparisons, corrected significant p-value < 0.025) (Fig. 1). We did not observe any significant correlations between changes in neurometabolite concentrations of myo-inositol, glx and glutamate in the ACC and changes in resting-state functional connectivity (between the pregenual ACC and other brain regions) between treatment groups.

### Group independent component analysis (ICA)

**Fig. 2:**
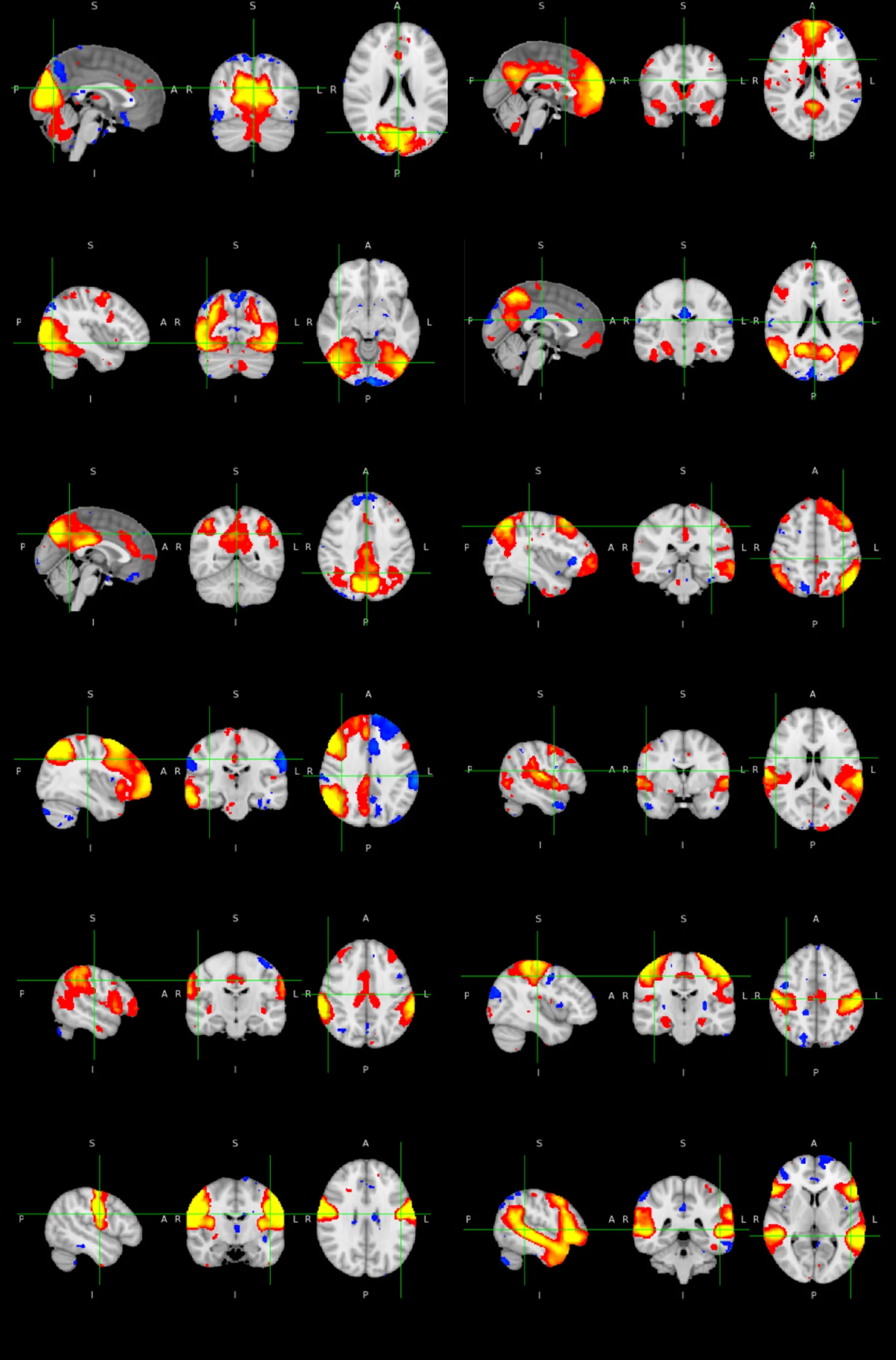

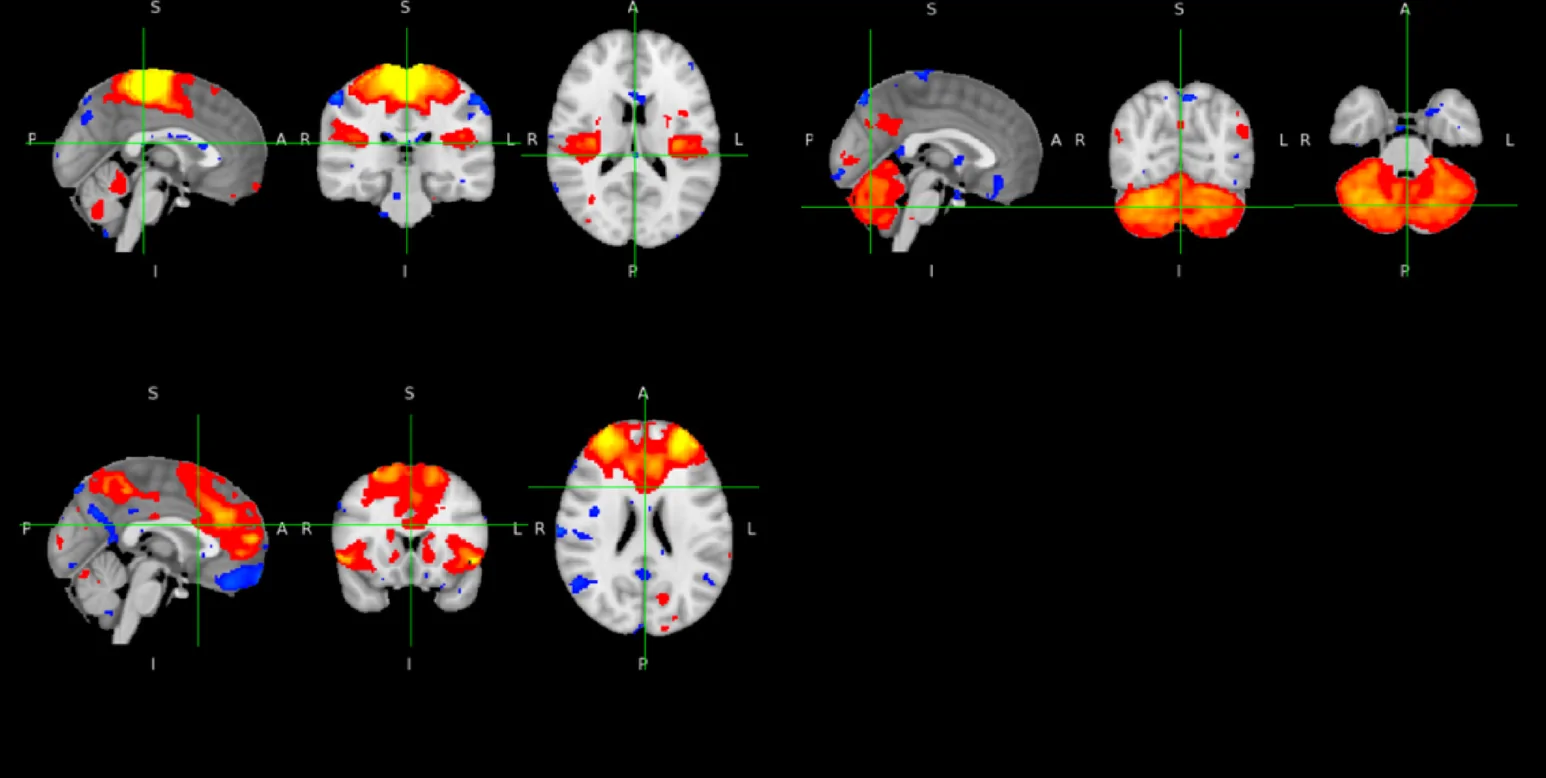
Resting state networks identified from group independent component analysis in axial, coronal and sagittal slices (*n* = 45). Threshold value of 2.3. Abbreviations: ECN: executive control network; DMN: default mode network; FPN: frontoparietal network; MNI: Montreal Neurological Institute.

Nineteen components were identified as resting-state networks (RSNs) of interest corresponding to previously identified canonical RSNs^27,34,37^, and six components were identified as noise (physiological and head movement). All the identified RSNs are presented in Fig. 2.

**Fig. 3:**
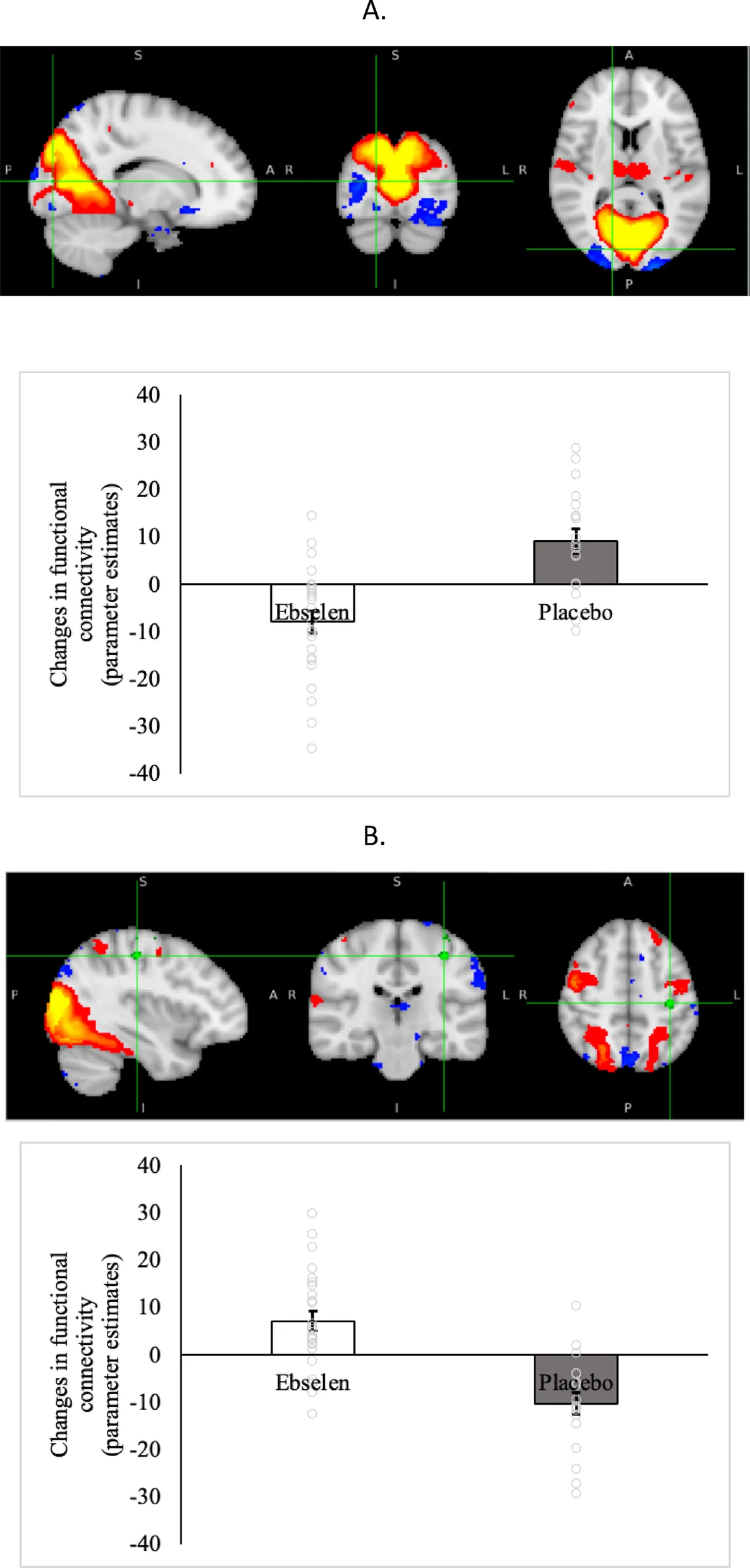

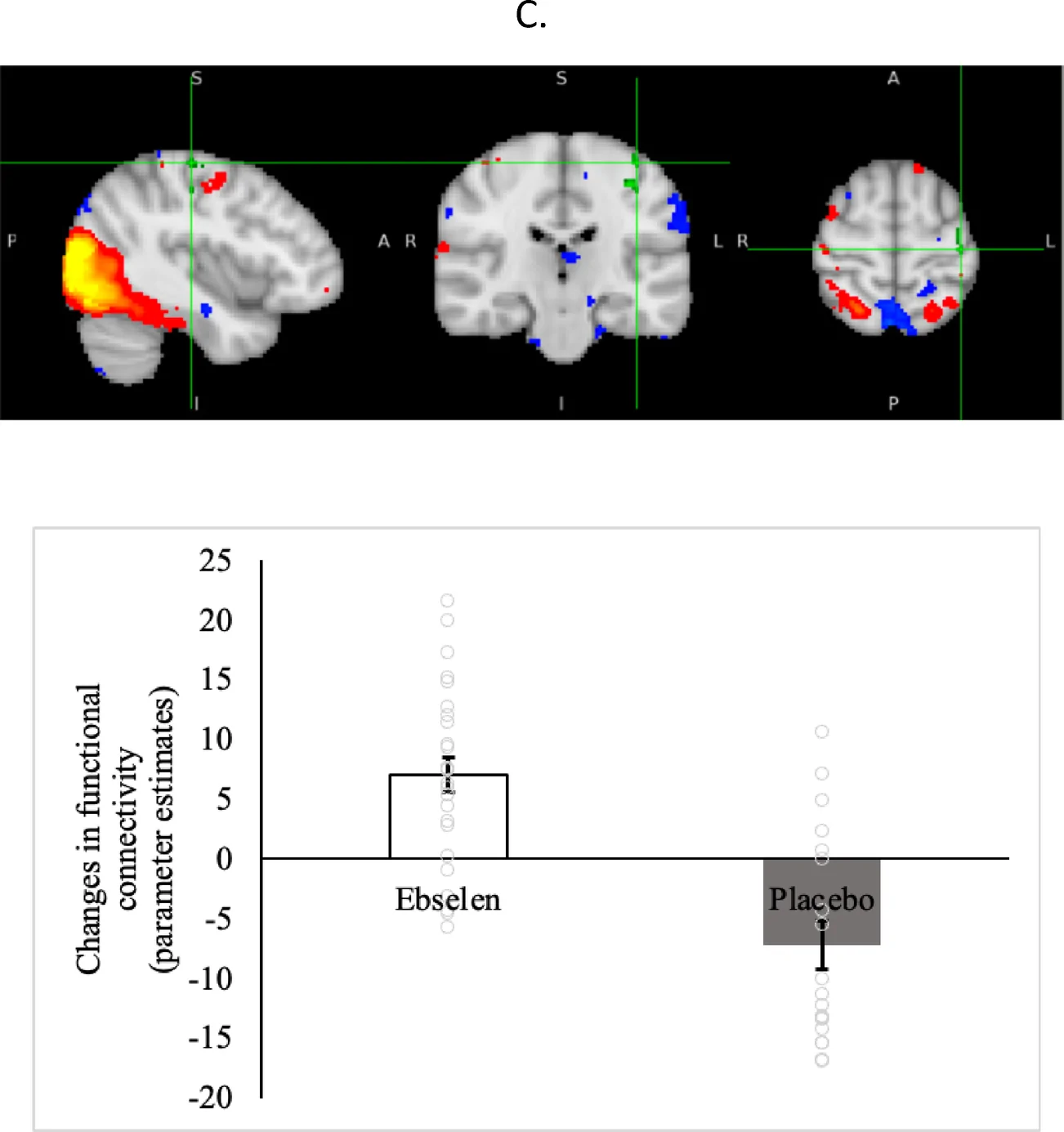
Changes in resting-state functional connectivity within the visual network and between visual and sensorimotor networks. Results are reported using TFCE method, with clusters considered significant at a family-wise error-controlled threshold of *p* < 0.05. The results were not significant after correction for multiple comparisons (19 RSNs and 2 *t-test*, corrected significant *p* < 0.001). A. Greater decrease in resting-state functional connectivity in the ebselen group than in the placebo group within the visual networks before adjustment. B & C. Greater increases in resting-state functional connectivity in the ebselen group than in the placebo group between visual and sensorimotor networks. (B: green colour, cluster size = 30 significant voxels, cursor position, MNI 63, 51, 61, uncorrected peak p-value − 0.008, t = 5.34; C: green colour, cluster size = 25 significant voxels, cursor position, MNI 65, 52, 67, uncorrected p-value − 0.025, t = 4.56). Note: Red-yellow: regions with correlated BOLD signal time-course; blue: regions with anti-correlated BOLD signal time-course; green: group differences.

Group comparisons, using TFCE method, with clusters considered significant at a family-wise error–controlled threshold of *p* < 0.05, revealed significant treatment effects within visual networks (number of significant voxel = 1, peak voxel MNI 37, 20, 41, uncorrected peak p-value 0.049, Hedge’s g=−1.481 (large effect size) and between visual networks and sensorimotor networks (Cluster 1: number of significant voxels = 30, peak voxel MNI 63, 51, 61, uncorrected peak p-value 0.008, Hedge’s g = 1.696 (large effect size); Cluster 2: number of significant voxels = 25, peak voxel MNI 65, 52, 67, uncorrected peak p-value 0.025, Hedge’s g = 1.731 (large effect size) (Fig. 3; Table 2). However, the effects did not survive correction with multiple RSNs (19 RSNs x 2 *t*-tests comparisons, corrected significant p-value < 0.001).

**Table 2:** Changes in resting-state functional connectivity in the ebselen and placebo groups after 7 days of treatment within visual networks and between visual and sensorimotor networks [*n* = 45]. Results are reported using TFCE method, family-wise error-controlled for ICA, with clusters considered significant at a threshold of *p* < 0.05. The results were not significant after correction for multiple comparisons with RSNs and *t-tests*.

Reference network	Network with significant voxel	Cluster size (number of voxels)	Peak *p*-value	Peak voxel (MNI coordinates)	Contrast
Seed-based analysis					
Pregenual ACC	Cerebellum	4	0.041	27, 41, 21	Ebselen > Placebo
Group ICA					
Visual	Somatosensory area (cluster 1)	30	0.008	63, 51, 61	Ebselen > Placebo
Visual	Somatosensory area (cluster 2)	25	0.025	65, 52, 67	Ebselen > Placebo
Visual	Visual area	1	0.049	37, 20, 41	Ebselen < Placebo

## Discussion

A short-term ebselen treatment increased changes in resting-state functional connectivity between the pregenual ACC and cerebellum, as well as between visual and sensorimotor networks but reduced changes in functional connectivity within the visual cortex. However, the effects did not survive correction for multiple comparisons. In addition, no significant correlations were observed between changes in ACC neurochemical concentrations over treatment course and a change in resting-state functional connectivity in the pregenual ACC. Negligible findings might be attributable to several factors, including the short treatment duration, heterogeneity in concurrent antidepressant regimens, small sample size, and the distinct clinical characteristics of this depression cohort.

Although short-term ebselen administration has been shown to induce significant neurochemical changes in the same cohort^13^, a longer duration of ebselen treatment might be required to elicit substantial changes in large-scale brain functional connectivity. While there are lines of evidence supporting changes in functional connectivity by antidepressants after 3–5 h, the studies reporting these findings typically involved healthy volunteers or unmedicated major depressive disorder patients^38,39^. Because of this, these studies are not directly comparable to our study, in which participants had chronic depression with inadequate response to ongoing antidepressant treatment. As such, it remains unknown whether ebselen may produce detectable changes in resting-state functional connectivity in unmedicated depressed patients. These differences should be considered when interpreting the results and further evaluated in future studies.

The divergent effects of ebselen on emotion processing observed between healthy volunteers^1,6^ and our current cohort^13^ may partly explain the negligible effects of ebselen on resting-state functional connectivity in our patient cohort. Prior studies in healthy volunteers reported that ebselen induced a positive emotional bias shift in the facial expression recognition task^1,6^ but not in other emotional processing domains, such as emotion encoding and memory. In contrast, ebselen did not induce any changes in emotional processing in our current depressed patient cohort^13^. Further, our cohort of depressed individuals, compared to healthy volunteers, showed no significant difference in facial expression recognition, but exhibited negative biases in emotional encoding and memory^40^. Additionally, a short-term lithium treatment in healthy volunteers produced a significant bias shift in the emotional encoding domain, suggesting the differing effects between lithium and ebselen^41^. The lack of the impact of ebselen on both emotional processing^13^ and resting-state functional connectivity in the examined cohort may be interpreted as convergent findings, suggesting that short-term ebselen treatment may not sufficiently engage the relevant neural circuits in the cohort of depressed individuals who do not respond to antidepressant treatment.

Given that ebselen is considered a lithium-mimetic agent, it is informative to consider the literature on the lithium’s effects on resting-state functional connectivity. It is also possible that our cohort included patients who were relatively non-responsive to ebselen, thereby reducing the power to detect treatment-related differences. Supporting this possibility, a previous study has shown that lithium responders and non-responders exhibit distinct brain activations patterns, with responders exhibiting higher activation within frontal and cingulate regions during a task-based fMRI paradigm involving emotional processing^42^. Similar variability may also be relevant to ebselen responsiveness. However, as ebselen has not yet been evaluated over a treatment duration sufficient to establish clinical response in depressed patients, this possibility remains speculative. Future longer-duration studies incorporating clinical outcomes and potential markers of ebselen responsiveness may help clarify whether treatment-related changes in functional connectivity are more detectable in responsive subgroups.

Although ebselen and lithium share overlapping mechanisms of actions, they likely exert their effects through partially distinct neurological pathways. Both agents can influence glutamatergic neurotransmission. However, the underlying mechanisms are different. Ebselen appears to modulate glutamatergic signalling by reducing synaptic glutamate levels through inhibition of glutaminase^4,43^, while lithium is thought to attenuate glutamatergic transmission by inhibiting N-methyl-D-aspartate (NMDA) receptor-mediated calcium influx^44^. Moreover, ebselen exhibits greater potency than lithium in inhibiting IMPase and shows higher selectivity for IMPase relative to glycogen synthase kinase-3ß^2^, suggesting important pharmacodynamic differences between the two agents.

Several limitations should be acknowledged. These include small sample size, lack of baseline fMRI data prior to initiation of standard antidepressant treatment, and short treatment duration. The sample size calculation was based on the primary behavioural outcome of the larger study, namely emotional processing, rather than on resting-state functional connectivity. The modest sample size may also have limited statistical power to detect more subtle effects of ebselen on functional connectivity and may have contributed to instability in the estimated effect sizes. Additionally, the lack of a healthy volunteer comparison group limits direct comparisons between disease and non-disease states. Also, participants were receiving a range of concurrent antidepressant medications, and medication class was not included as a covariate in the analyses. This heterogeneity may have introduced variability in baseline neural activity and reduced sensitivity to detect additional pharmacological effects of ebselen. Moreover, although ebselen has been shown to affect inositol and glutamate concentrations, the present study did not include pharmacokinetic or direct target engagement measures, limiting our ability to confirm the extent of central target engagement during the treatment period. Furthermore, although exploratory analyses suggested changes within visual networks and between visual and sensorimotor networks, these findings did not survive correction for multiple comparisons. As such, they should be interpreted with caution and may reflect Type I error rather than meaningful neurobiological effects. Alternatively, given the involvement of visual and sensorimotor networks in perceptual and integrative processing, further studies would be required to determine whether these observations represent reproducible treatment-related effects. Also, the study lacked the baseline group comparisons. While the randomised allocation together with the absence of significant differences in demographic and clinical baseline measures lessen concern regarding systematic baseline imbalance, such analysis could provide additional context for the interpretation of longitudinal rsFC findings.

Nevertheless, to our knowledge, this is the first study to examine the effects of ebselen on resting-state functional connectivity in a clinical population. As such, despite the largely negative findings, this study contributes novel and valuable insights to the growing body of ebselen research and informs the designs of future studies investigating lithium-mimetic agents in mood disorders. Future studies should prioritise larger, adequately powered samples with longer treatment durations, stratification by treatment response, and the inclusion of unmedicated depressed patients or healthy control groups. These designs would allow for better elucidation of the neurofunctional effects of ebselen and its potential role as a mood-stabilising or antidepressant agent.

Acute ebselen treatment as an add-on therapy to standard antidepressants did not produce significant changes in resting-state functional connectivity in depressed patients who had inadequate response to standard antidepressant treatment. Although ebselen showed effects on resting-state functional connectivity between the pregenual ACC and the cerebellum, as well as within visual network and between visual and sensorimotor networks, these findings did not survive correction for multiple comparisons. These findings may reflect limitations related to the short treatment duration, small and heterogenous sample, and the complexity of treatment-resistant depression.

## Data Availability

The datasets generated during and/or analysed during the current study are available from the corresponding author on reasonable request.

## References

[1] Singh, N. et al. Effect of the putative lithium mimetic ebselen on brain myo-inositol, sleep, and emotional processing in humans. *Neuropsychopharmacology* **41**, 1768–1778 (2016). 26593266 10.1038/npp.2015.343 PMC4770517

[2] Singh, N. et al. A safe lithium mimetic for bipolar disorder. *Nat. Commun.* **4**, 1332 (2013). 23299882 10.1038/ncomms2320 PMC3605789

[3] Sharpley, A. L. et al. A phase 2a randomised, double-blind, placebo-controlled, parallel-group, add-on clinical trial of ebselen (SPI-1005) as a novel treatment for mania or hypomania. *Psychopharmacol. (Berl)*. **237**, 3773–3782 (2020). 10.1007/s00213-020-05654-1 PMC7683468 32909076

[4] Ramli, F. F., Cowen, P. J. & Godlewska, B. R. The potential use of ebselen in treatment-resistant depression. *Pharmaceuticals (Basel)*. **15**, 485 (2022). 35455482 10.3390/ph15040485 PMC9030939

[5] Masaki, C. et al. Effects of the potential lithium-mimetic, ebselen, on brain neurochemistry: A magnetic resonance spectroscopy study at 7 tesla. *Psychopharmacol. (Berl)*. **233**, 1097–1104 (2016). 10.1007/s00213-015-4189-2 PMC4759215 26758281

[6] Masaki, C. et al. Effects of the potential lithium-mimetic, ebselen, on impulsivity and emotional processing. *Psychopharmacol. (Berl)*. **233**, 2655–2661 (2016). 10.1007/s00213-016-4319-5 PMC4917572 27256357

[7] Antoniadou, I. et al. Ebselen has lithium-like effects on central 5-HT(2A) receptor function. *Br. J. Pharmacol.* **175**, 2599–2610 (2018). 29488218 10.1111/bph.14179 PMC6003638

[8] Antoniadou, I. et al. (eds) Effect of ebselen, a putative lithium mimetic, on central 5-HT2C receptor function in the mouse. *Proceedings of the British Pharmacological Society*. (2015).

[9] Sun, J. et al. Distinct patterns of functional brain network integration between treatment-resistant depression and non treatment-resistant depression: A resting-state functional magnetic resonance imaging study. *Prog Neuropsychopharmacol. Biol. Psychiatry*. **120**, 110621 (2023). 36031163 10.1016/j.pnpbp.2022.110621

[10] Siegel, J. S. et al. Prolonged ketamine infusion modulates limbic connectivity and induces sustained remission of treatment-resistant depression. *Psychopharmacol. (Berl)*. **238**, 1157–1169 (2021). 10.1007/s00213-021-05762-6 PMC7969576 33483802

[11] Wade, B. S. C. et al. Anterior default mode network and posterior insular connectivity is predictive of depressive symptom reduction following serial ketamine infusion. *Psychol. Med.* **52**, 2376–2386 (2022). 35578581 10.1017/S0033291722001313 PMC9527672

[12] Su, T. P. et al. Low-dose ketamine improved brain network integrity among patients with treatment-resistant depression and suicidal ideation. *Psychiatry Res.* **345**, 116377 (2025). 39889566 10.1016/j.psychres.2025.116377

[13] Ramli, F. F. et al. Effects of ebselen addition on emotional processing and brain neurochemistry in depressed patients unresponsive to antidepressant medication. *Transl Psychiatry*. **14**, 200 (2024). 38714646 10.1038/s41398-024-02899-8 PMC11076504

[14] Harmer, C. J., Shelley, N. C., Cowen, P. J. & Goodwin, G. M. Increased positive versus negative affective perception and memory in healthy volunteers following selective serotonin and norepinephrine reuptake inhibition. *Am. J. Psychiatry*. **161**, 1256–1263 (2004). 15229059 10.1176/appi.ajp.161.7.1256

[15] Brant-Zawadzki, M., Gillan, G. D. & Nitz, W. R. MP RAGE: A three-dimensional, T1-weighted, gradient-echo sequence–initial experience in the brain. *Radiology* **182**, 769–775 (1992). 1535892 10.1148/radiology.182.3.1535892

[16] Shah, S. et al. (eds) Rapid fieldmap estimation for cardiac shimming. *Proc Intl Soc Mag Reson Med*. (2009).

[17] Deelchand, D. K. et al. Two-site reproducibility of cerebellar and brainstem neurochemical profiles with short-echo, single-voxel MRS at 3T. *Magn. Reson. Med.* **73**, 1718–1725 (2015). 24948590 10.1002/mrm.25295 PMC4272339

[18] Jenkinson, M., Beckmann, C. F., Behrens, T. E., Woolrich, M. W. & Smith, S. M. *FSL Neuroimage* **62**, 782–790 (2012). 21979382 10.1016/j.neuroimage.2011.09.015

[19] Beckmann, C. F., DeLuca, M., Devlin, J. T. & Smith, S. M. Investigations into resting-state connectivity using independent component analysis. *Philos. Trans. R Soc. Lond. B Biol. Sci.* **360**, 1001–1013 (2005). 16087444 10.1098/rstb.2005.1634 PMC1854918

[20] Sankar, A. et al. Altered frontal cortex functioning in emotion regulation and hopelessness in bipolar disorder. *Bipolar Disord*. **23**, 152–164 (2021). 32521570 10.1111/bdi.12954 PMC7790437

[21] Griffanti, L. et al. Hand classification of fMRI ICA noise components. *Neuroimage* **154**, 188–205 (2017). 27989777 10.1016/j.neuroimage.2016.12.036 PMC5489418

[22] Masaki, C. et al. Effects of the potential lithium-mimetic, ebselen, on brain neurochemistry: a magnetic resonance spectroscopy study at 7 tesla. *Psychopharmacol. (Berl)*. **233**, 1097–1104 (2016). 10.1007/s00213-015-4189-2 PMC4759215 26758281

[23] Singh, N. et al. Effect of the putative lithium mimetic ebselen on brain myo-inositol, sleep, and emotional processing in humans. *Neuropsychopharmacology* **41**, 1768–1778 (2016). 26593266 10.1038/npp.2015.343 PMC4770517

[24] Altinay, M., Karne, H. & Anand, A. Lithium monotherapy associated clinical improvement effects on amygdala-ventromedial prefrontal cortex resting state connectivity in bipolar disorder. *J. Affect. Disord*. **225**, 4–12 (2018). 28772145 10.1016/j.jad.2017.06.047 PMC5844774

[25] Rolls, E. T., Huang, C. C., Lin, C. P., Feng, J. & Joliot, M. Automated anatomical labelling atlas 3. *Neuroimage* **206**, 116189 (2020). 31521825 10.1016/j.neuroimage.2019.116189

[26] Beckmann, C. F., Mackay, C. E., Filippini, N. & Smith, S. M. Group comparison of resting-state FMRI data using multi-subject ICA and dual regression. *NeuroImage* **47**, S148 (2009).

[27] Martens, M. A. G., Filippini, N., Harmer, C. J. & Godlewska, B. R. Resting state functional connectivity patterns as biomarkers of treatment response to escitalopram in patients with major depressive disorder. *Psychopharmacol. (Berl)*. **239**, 3447–3460 (2022). 10.1007/s00213-021-05915-7 PMC9584978 34477887

[28] Filippini, N. et al. Distinct patterns of brain activity in young carriers of the APOE-epsilon4 allele. *Proc. Natl. Acad. Sci. U S A*. **106**, 7209–7214 (2009). 19357304 10.1073/pnas.0811879106 PMC2678478

[29] Douaud, G. et al. Anatomically related grey and white matter abnormalities in adolescent-onset schizophrenia. *Brain* **130**, 2375–2386 (2007). 17698497 10.1093/brain/awm184

[30] Good, C. D. et al. A voxel-based morphometric study of ageing in 465 normal adult human brains. *Neuroimage* **14**, 21–36 (2001). 11525331 10.1006/nimg.2001.0786

[31] Smith, S. M. et al. Advances in functional and structural MR image analysis and implementation as FSL. *Neuroimage* **23** (Suppl 1), S208–S219 (2004). 15501092 10.1016/j.neuroimage.2004.07.051

[32] Nichols, T. E. & Holmes, A. P. Nonparametric permutation tests for functional neuroimaging: a primer with examples. *Hum. Brain Mapp.* **15**, 1–25 (2002). 11747097 10.1002/hbm.1058 PMC6871862

[33] Smith, S. M. & Nichols, T. E. Threshold-free cluster enhancement: addressing problems of smoothing, threshold dependence and localisation in cluster inference. *Neuroimage* **44**, 83–98 (2009). 18501637 10.1016/j.neuroimage.2008.03.061

[34] Smith, S. M. et al. Correspondence of the brain’s functional architecture during activation and rest. *Proc. Natl. Acad. Sci. U S A*. **106**, 13040–13045 (2009). 19620724 10.1073/pnas.0905267106 PMC2722273

[35] Provencher, S. W. Estimation of metabolite concentrations from localized in vivo proton NMR spectra. *Magn. Reson. Med.* **30**, 672–679 (1993). 8139448 10.1002/mrm.1910300604

[36] Near, J. et al. Preprocessing, analysis and quantification in single-voxel magnetic resonance spectroscopy: Experts’ consensus recommendations. *NMR Biomed.* **34**, e4257 (2021). 32084297 10.1002/nbm.4257 PMC7442593

[37] Yeo, B. T. et al. The organization of the human cerebral cortex estimated by intrinsic functional connectivity. *J. Neurophysiol.* **106**, 1125–1165 (2011). 21653723 10.1152/jn.00338.2011 PMC3174820

[38] Cheng, Y. et al. Resting-state brain alteration after a single dose of SSRI administration predicts 8-week remission of patients with major depressive disorder. *Psychol. Med.* **47**, 438–450 (2017). 27697079 10.1017/S0033291716002440

[39] Schaefer, A. et al. Serotonergic modulation of intrinsic functional connectivity. *Curr. Biol.* **24**, 2314–2318 (2014). 25242032 10.1016/j.cub.2014.08.024

[40] Ramli, F. F. et al. Negative bias in encoding and recall memory in depressed patients with inadequate response to antidepressant medication. *Psychopharmacology (Berl)* 10.1007/s00213-025-06857-0 (2025). 40643613 PMC12979351

[41] Ramli, F. F., Harmer, C. J., Cowen, P. J. & Godlewska, B. R. Lithium effects on impulsivity and emotional processing. *Sci. Rep.* **15**, 45216 (2025). 41276636 10.1038/s41598-025-29216-7 PMC12749009

[42] Rootes-Murdy, K. et al. A pilot fMRI study of lithium response in bipolar disorder. *Psychiatry Res. Neuroimaging*. **286**, 1–3 (2019). 30822677 10.1016/j.pscychresns.2019.02.003 PMC6749831

[43] Mota, F. et al. Investigating the effects of ebselen, a potential new lithium mimetic, on glutamate transmission. *Synapse* **74**, e22151 (2020). 32056277 10.1002/syn.22151

[44] Nonaka, S., Hough, C. J. & Chuang, D. M. Chronic lithium treatment robustly protects neurons in the central nervous system against excitotoxicity by inhibiting N-methyl-D-aspartate receptor-mediated calcium influx. *Proc Natl Acad Sci U S A* **95**, 2642–7 (1998). 9482940 10.1073/pnas.95.5.2642 PMC19446

